# Progressive Cellularization of Blastoderm and Extraembryonic Tissue Formation in the Ant *Camponotus floridanus*

**DOI:** 10.1002/jez.b.70028

**Published:** 2026-06-14

**Authors:** Nihan Sultan Milat, Murat Pekmez, Ab. Matteen Rafiqi

**Affiliations:** ^1^ Department of Molecular Biology and Genetics, Institute of Graduate Studies in Sciences Istanbul University Istanbul Türkiye; ^2^ Beykoz Institute of Life Sciences and Biotechnology Bezmialem Vakıf University Istanbul Türkiye; ^3^ Department of Molecular Biology and Genetics Faculty of Science, Istanbul University Istanbul Türkiye

**Keywords:** amnion, ants, blastoderm, cellularization, serosa, trophocytes

## Abstract

Blastoderm formation represents a key transition from a syncytial to a cellular embryo and provides the basis for subsequent embryonic patterning in insects. In most insect models, this transition occurs through synchronous cellularization, producing a uniform blastoderm that is patterned only afterward. Whether this sequence represents a common developmental principle across insects remains unclear. Here, we show that in the carpenter ant *Camponotus floridanus*, cellularization proceeds progressively rather than synchronously and establishes spatially differentiated blastoderm domains. Cellularization initiates at the anterior and progresses posteriorly, while a second front from the posterior advances in the opposite direction. These opposing fronts converge at the site where the germline capsule subsequently forms. At the same time, a regionalized blastoderm rather than a uniform blastoderm is established. The extraembryonic tissues amnion, serosa, and trophocytes emerge along progressive cellularization through coordinated morphogenetic dynamics and exhibit a distinct mode of organization. Our data present a revised understanding of cellularization of blastoderm in insects and broaden the comparative framework of embryogenesis described in model organisms.

## Graphical abstract

Illustration of the process of cellularization in *Camponotus floridanus* (top row) compared to Drosophila melanogaster (bottom row). Progressive direct formation of differentiating blastoderm mode is depicted using hexagons that represent energids (without borders) and cells (with borders). Colors of nuclei represent different cell types.

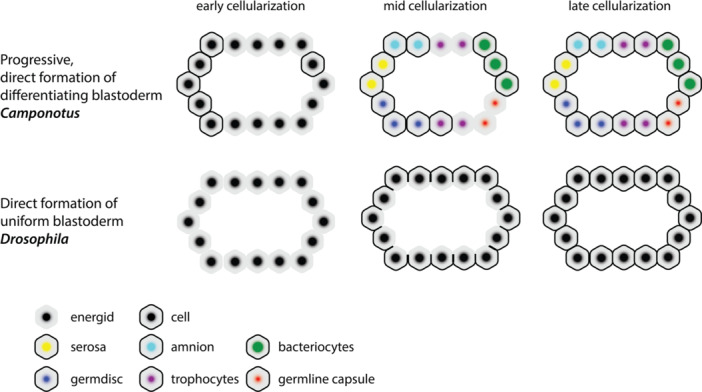

## 1 Introduction

Cellularization is a crucial process in embryogenesis, marking the transition from a multinucleated syncytium to a cellular blastoderm in insects. This process, in which membranes form around individual nuclei and cytoplasm surrounding them (termed ‘energids’) to convert the syncytium into a cellularized embryo, has been extensively studied for decades in multiple insect groups (Benton et al. 2013; Donoughe et al. 2022; Handel et al. 2000; Ho et al. 1997; Kelly and Huebner 1989; Nakao 2021; Sarashina et al. 2005; Van Der Zee et al. 2015). These studies have shown that while cellularization is broadly conserved, its dynamics and outcomes vary substantially among lineages. Two longstanding modes of blastoderm formation have been described: ‘the direct formation of a uniform blastoderm’, in which cleavage energids distribute evenly across the periplasm and cellularization produces a homogeneous epithelial layer (Anderson 1972a; Sarashina et al. 2005; Tojo and Machida 1998), and ‘the direct formation of a differentiating blastoderm’, in which cellularization coincides with the establishment of differential cell densities and early tissue heterogeneity (Handley et al. 1998; Nakao 2021; Sander 1976). This framework underscores that cellularization can serve not only to partition nuclei into individual cells but also to be the initial step in tissue differentiation at the blastoderm stage.

How cellularization interfaces with tissue morphogenesis can be studied particularly well using extraembryonic (EE) tissues, because their remarkable structural diversity and accessibility make them an ideal system for investigating early developmental processes (Panfilio 2008). In insects in general, the amnion covers the ventral side of the developing embryo and encloses an amniotic cavity, while the serosa envelops the embryo, amnion, and yolk (Farnesi et al. 2015; Jacobs et al. 2013; Jacobs et al. 2014; Rezende et al. 2008). Although this organization is widespread across insects, notable variations exist (Dorn 1976; Ikeda and Machida 2001; Jura 1972; Machida 2006; Uemiya and Ando 1991). In Hymenoptera, the trajectory of EE tissue development also varies between lineages (Anderson 1972a; Bull 1982; Fleig and Sander 1986; Ivanova‐Kasas 1959; Shafiq 1954). In basal lineages of Hymenoptera, such as sawflies, the amnion forms but breaks down at an early stage and remains only in a rudimentary state. In later stages, dorsal closure takes place within a continuous serosa in the absence of amnion. Serosa persists till the hatching and secretes a cuticle (Ivanova‐Kasas 1959; Shafiq 1954). In contrast, higher Hymenoptera, including bees and wasps, generally lack a ventral amniotic cavity, and most retain a transient dorsal amnion that extends over the yolk rather than surrounding the embryo (Anderson 1972b; Bull 1982; Fleig and Sander 1988).

The recent comprehensive developmental tables of the carpenter ant *C. floridanus*, the Pharaoh ant *Monomorium pharaonis*, as well as descriptions of embryogenesis in other ants such as *Atta texana, Mycocepurus smithii*, and *Solenopsis invicta* have provided an essential framework for understanding embryonic and EE organization in ant species (Formicidae) (Chen et al. 2025; Fang et al. 2025; Rajakumar et al. 2024). However, the developmental tables and the embryonic descriptions are focused on general events in embryogenesis and rely mainly on fixed material, leaving the early emergence, cellular origins, and live morphogenetic behaviors of the specific EE tissues—as well as the dynamic processes underlying blastoderm formation and early extraembryonic morphogenesis—largely uncharacterized. In *C. floridanus*, EE tissues develop differently, likely to distance the embryo from bacteria, alongside embryonic development that is radically different from that of other ants and insects (Rafiqi et al. 2020; Rajakumar et al. 2024). Insect embryos typically form at the posterior‐ventral or throughout the entire egg. In *C. floridanus*, the embryo forms in the anterior‐ventral region while bacteria occupy the posterior pole of the eggs within the bacteriocytes. In *C. floridanus*, as in *A. mellifera* and *Nasonia vitripennis*, the amnion originates from the dorsal side but later expands to enclose the yolk while the serosa originates in the anterior of the germ disc and forms a continuous outer epithelium that persists until hatching (Bull 1982; Chen et al. 2025; Fleig and Sander 1988). In *M. pharaonis*, the EE tissue originates on the dorsal anterior of the egg (Rajakumar et al. 2024). However, unlike the previously described wasps, bees, or the ant species, in *C. floridanus*, the amnion and serosa also cover the bacteriocytes and the germline capsule containing germline nuclei and bacteria (Chen et al. 2025; Durmuş et al. 2025; Rafiqi et al. 2020). Additionally, external to the amnion and underneath the serosa, a third EE cell type – the trophocytes – develops. The trophocytes are nutritive cells commonly referred to as fat cells, which are incorporated into embryonic tissues through resorption or are fed upon by the pre‐larvae at later stages (Chen et al. 2025; Danyk and Mackauer 1996; Tanquary 1913).

Here, we investigate the cellular dynamics of embryonic blastoderm development in *C. floridanus*. Aided by morphological characterization and live imaging, we investigate the mode of cellularization and its effect on the subsequent emergence of EE tissues within the blastoderm. We also explore morphogenetic behaviors for each EE tissue, focusing on the diversity of EE movements. Our results describe blastoderm formation and EE development in *C. floridanus* and compare it with previously described direct and indirect formation of differentiated blastoderm.

## 2 Results

### 2.1 Direct Formation of Differentiated Blastoderm in *C. floridanus*

**Figure 1:**
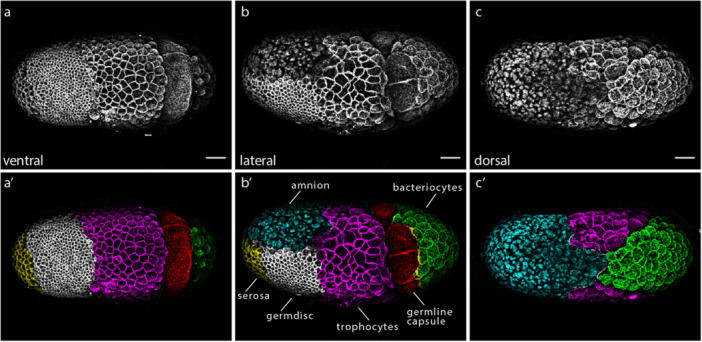
Representative images showing the blastoderm stage of *C. floridanus*. (a) Ventral, (b) lateral, and (c) dorsal views of embryos from representative time points of live imaging at the blastoderm stage. (a′–c′) Different tissues are color‐coded for clarity: the serosa (yellow), germ disc (gray), amnion (cyan), trophocytes (magenta), germline capsule (red), and bacteriocytes (green). Each column represents a different embryo imaged from a distinct orientation. Anterior is to the left. Scale bar = 50 µm.

To visualize the blastoderm cells in *C. floridanus*, we injected LifeAct‐mCherry fusion protein into early embryos and imaged them at the blastoderm stage. It enabled the visualization of dynamic cortical actin rearrangements that precede cellularization. The *C. floridanus* embryonic blastoderm is made up of at least six distinctly patterned cells around the periphery of the egg (Chen et al. 2025). Using LifeAct‐mCherry, we were able to distinguish and follow the development of all of these cell types (Figure 1 and Embedded Video 1). Germdisc cells, located anterior–ventrally, were relatively small and polygonal, typically pentagonal in outline (Figure 1a,a',b,b'). Serosa cells were anterior to the germdisc and resembled germdisc cells in shape but were significantly larger (13 µm *vs.* 8 µm, *n* = 20, *p* = 7×10^−9^ (Figure 1a,a',b,b'). Immediately posterior to the serosa cells and dorsal to the germ disc, the amnion appeared as a granular region, not intensely stained with LifeAct. Furthermore, the anterior half of the amnion contained smaller, densely packed granules. In contrast, its posterior half appeared larger and loosely packed, such that anterior and posterior domains differed in morphology (Figure 1b,b',c,c'). In Hoechst‐stained fixed embryos, the amnion consisted of a region with the least density of nuclei compared to other tissues, which we interpret as 8–10 highly stretched amnion cells (Figure 3). However, since the amnion in later stages consists of a much higher number of cells, we suspect cells bordering these to also be of amnion identity. Trophocytes occupied the middle barrel‐shaped region between the dorsal amnion and the ventral germdisc and the germline capsule. The trophocytes were conspicuously larger than other blastoderm cells; while also polygonal in shape, they varied in outline and size but were readily distinguished by their overall greater size (20 µm, *n* = 20, *p* = 2×10^−12^) (Figure 1a–c'). Bacteriocytes occupied the posterior domain of the embryo and resembled trophocytes in size and morphology (Figure 1a–c'). The trophocytes and bacteriocytes could easily be distinguished using DNA stain DAPI, which strongly stains the bacteria inside bacteriocytes (Chen et al. 2025). Finally, at the interface between the bacteriocyte region and the EE domains, germline capsule cells appear as the largest blastodermal cells observed (Figure 1a,a',b,b'). Therefore, *C. floridanus* blastoderm embryos contain six distinguishable cell types, which are foreshadowed by differences in nuclear density, cytoplasmic granularity, presence of bacteria, and distances between cell–cell boundary initiation. This implies that this species undergoes non‐uniform blastoderm formation and resembles the direct formation of a differentiating blastoderm described in other insects (Handley et al. 1998; Nakao 2021; Sander 1976).

### 2.2 Progressive Cellularization Along the Anterior‐Posterior Axis in *C. floridanus*

**Figure 2:**
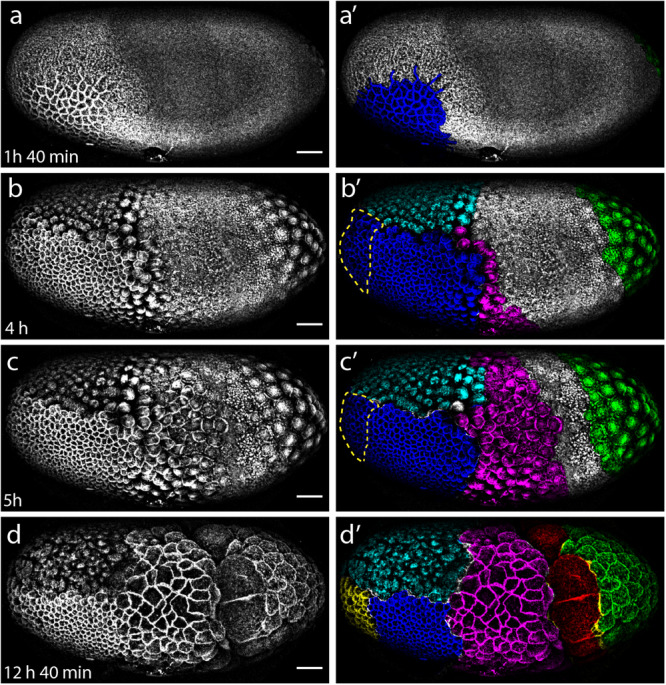
Progressive cellularization shapes the spatial organization of tissues in *C. floridanus*. (a–d′) Snapshots from time‐lapse imaging of a *C. floridanus* embryo injected with LifeAct–mCherry. (a, a′) The first phase of cellularization originates from the ventral‐anterior region and forms the germ disc and presumptive serosa. (b, b′) The second anterior phase extends into the amnion and trophocytes, while the posterior phase begins within the bacteriocyte region. (c, c′) Cellularization progresses to the amnion, trophocytes, and bacteriocytes. (d, d′) Finally, the anterior and posterior phases converge, initiating cellularization of the germline capsule. At this stage, the serosa can be distinguishable from the germ disc. The yellow dashed lines indicate the approximate region where presumptive serosa forms (b’,c’). Different tissues are color‐coded for clarity: germ disc (blue), amnion (cyan), trophocytes (magenta), bacteriocytes (green), serosa (yellow), and germline capsule (red). Anterior is to the left, and dorsal is up. Scale bar = 50 µm.

To trace the dynamics of the blastoderm stage in *C. floridanus*, we performed time‐lapse imaging by using LifeAct‐mCherry protein injection in early embryos prior to the blastoderm stage. Most model insects are rapidly developing, ranging from 1 day in *Drosophila*, 3 days in *Tribolium*, to 6 days in *Gryllus* (Campos‐Ortega and Hartenstein 2013; Donoughe and Extavour 2016; Handel et al. 2000). Unlike these species, *C. floridanus* embryogenesis spans 15 days, limiting continuous time‐lapse imaging of complete embryogenesis. We, therefore, optimized our time of injection and imaging to capture stages when embryonic and EE structures are established. Our time‐lapse imaging unexpectedly demonstrated that cellularization occurred in multiple phases, initiating at the anterior‐ventral germdisc, and advancing in a dorsal–posterior direction, resulting in a gradual rather than uniform transition from a syncytial to a cellular blastoderm. In addition to this progression, independent cellularization was initiated in the posterior bacteriocyte domain, which advanced anteriorly. Finally, where these opposing fronts converged, the germline capsule formed (Figure 2, Supporting Figure S1 and Embedded Videos [Link], [Link], [Link]).

To understand how progressive cellularization influences tissue formation, we further analyzed the time‐lapse images in detail. Before cellularization, the embryo showed differences in cortical actin presence, high in the anterior as well as the future germline capsule (Supporting Figure S1a,b). Independent of the blastoderm cellularization, in *C. floridanus*, the pole cells cellularized earlier, which is consistent with data from other insects such as *Drosophila melanogaster* (Supporting Figure S1c) (Swanson and Poodry 1980). The initiation of the first phase of blastoderm cellularization occurred after pole cell formation. The first phase of anterior to posterior cellularization initiated in the anterior region fated to form the germdisc (Figure 2a,a', Supporting Figure S1d,d', and Embedded Video 1 0–2 s and Embedded Video 2). In the second phase of anterior to posterior cellularization, it progressed into the regions fated to become serosa and amnion. Subsequently, the trophocyte region underwent cellularization (Figure 2b,b', Supporting Figure S1f,g', and Embedded Video 1 2–4 s, andEmbedded Video 3). While the cellularization advanced from anterior to posterior, the bacteriocyte domain progressed from posterior to anterior direction (Figure 2b‐c', Supporting Figure S1f–i', Embedded Video 1 2–6 s, and Embedded Video 3). Finally, these cellularized regions converged at the prospective germline capsule region, initiating its cellularization, forming large cells (Figure 2d,d', Supporting Figure S1j,k'; and Embedded Video 1 5–10 s). However, the cells in this region subsequently fused to form the germline capsule. Together, these dynamics reveal that progressive cellularization in *C. floridanus* coincides with the gradual formation of distinct embryonic and EE tissues.

### 2.3 Morphogenetic Movements of the Extraembryonic Tissues in *C. floridanus*

**Figure 3:**
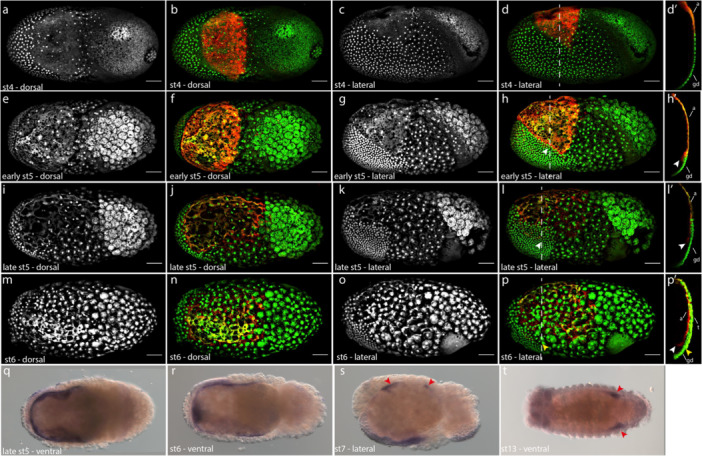
*Cfl*‐*piwi* expression marks the development of the amnion. (a–p) Confocal‐imaged and (q–t) DIC‐imaged *C. floridanus* embryos stained with a probe against *Cfl‐piwi*. (a–d) onset of cellularization stage 4, (e–h) early stage 5, (i–l, and q) late stage 5, (m–p, and r) stage 6, (s) stage 7, and (t) stage 13. (d’,h’,l’ and p’) show cross sections. Note that confocal‐imaged embryos are stained with Hoechst (a‐p) and shown in grayscale, except in merged panels where nuclei are shown in green and *piwi* expression in red (b,d,d’; f,h,h’; j,l,l’; n,p, p’). *piwi* expression is shown in DIC‐imaged embryos in purple (q–t). Cross‐sections are indicated by dashed lines in (d,h,l,p). White arrowheads indicate the leading edge of the amnion as it slips beneath the germ disc. The yellow arrowhead marks the boundary between the germ disc and trophocytes. Red arrows indicate *piwi* expression, highlighting diminished expression at stage 7 (s) and expression in the presumptive gonad at stage 13 (t). a, amnion; gd, germdisc; t, trophocytes. Fixed embryos were stage‐matched based on morphology according to Chen et al. (2025). Anterior is to the left, and dorsal is up.

**Figure 4:**
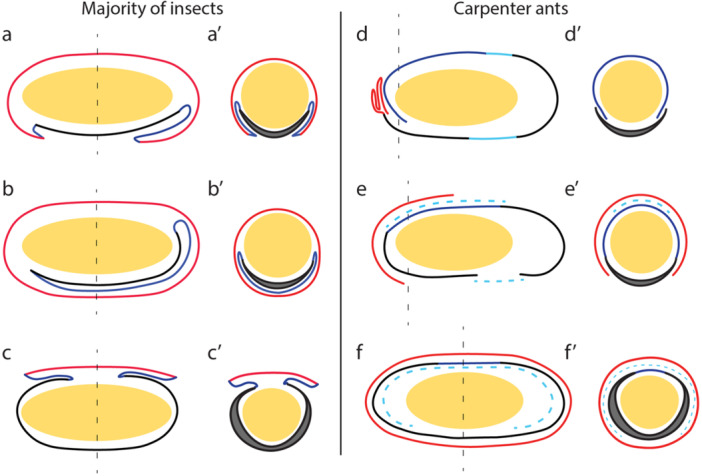
Illustration of extraembryonic membrane formation in *C. floridanus* compared to the known models. Schematic representation comparing the organization and morphogenetic behavior of extraembryonic membranes during embryogenesis in the majority of insects (a–c′) and in *C. floridanus* (d–f′). The three major developmental stages are shown: (a, d) blastoderm formation, (b, e) germband extension, and (c, f) dorsal closure. Each stage is additionally depicted in cross‐sectional views (a′–c′ for the generalized insect model, and d′–f′ for *C. floridanus*). Anterior is to the left, and dorsal is up. The germ band is shown in black, the amnion in dark blue, the serosa in red, the trophocytes in light blue, and the yolk in yellow. Dashed lines represent discontinuous tissue.

*C. floridanus* embryos contain three morphologically and behaviorally distinct EE tissues—the amnion, serosa, and trophocytes—that exhibit dynamic rearrangements during early development (Figure 3, Figure 4, Supporting Figure S2, and Embedded Videos [Link], [Link], [Link]). Among them, the amnion displayed highly dynamic and previously unreported movements. The amniotic cells consisted of a region with the least density of nuclei compared to other tissues, visible as highly stretched amnion cells (Figure 3a–o) and had less sharply defined actin‐enriched cell boundaries compared to their neighboring cells (Supporting Figure 1g,g',i, i',k,k'). The amnion starts to form during the second phase of cellularization, extending toward both the anterior and lateral directions, with a transient anterior movement followed by a predominant lateral expansion (Embedded Video 1 2–7 s). These movements suggest that the amnion follows a trajectory where it first contacts the germdisc domain (its anterior movement), then moves laterally, slipping between the germdisc and the yolk (its lateral movement) (Figure 3, Figure 4, and Embedded Video 4). The inward slipping of the amniotic fold is unlike that of other insects, where the it covers the surface of the germ disc ventrally (Panfilio 2008). This was followed by a posterior extension and the posterior half of the amnion appeared to push against adjacent trophocytes as it expanded posteriorly (Embedded Video 1 7–12 s). Although completion of a dorsal amniotic sheath was not captured at this stage, the amnion likely extends to a distal margin, which will ultimately enclose the dorsal of the yolk and subsequently the germline capsule and bacteriocyte domains at a later stage (Chen et al. 2025). Soon after posterior movement of amnion, the trophocytes were displaced from the posterior and lateral edges, then migrated onto the dorsal surface, where they converged along the midline from posterior to anterior, sealing over the amnion (Embedded Video 1 12–42 s and Embedded Video 5). Throughout this process, trophocytes exhibited strikingly dynamic behaviors, rotating, sliding over one another, and displaying remarkable flexibility. The erratic movements suggest that trophocytes cushion the spaces between the embryo, amnion, and serosa during late blastoderm stages.

The serosa cells did not follow the movements of the amnion; instead, their development appeared to initiate independently in the anterior region of the germ disc (Figure 2, Supporting Figure S1 and S2, Embedded Video 1 5 s to 1 min 3 s and Embedded Video 6). The serosa cells were difficult to distinguish due to their similar size and morphology to germdisc cells (Embedded Video 1 5–8 s). As development proceeded, the serosa became more distinct and could be reliably identified (Embedded Video 1 8–16 s). Live imaging indicates that the serosa maintains its position for a period, with no obvious displacement (Embedded Video 1 16–18 s). Consistent with this, in fixed material, serosa cells were observed folded at their original position. (Supporting Figure S2a–a'”). These cells clustered in a multilayered configuration at the anterior–dorsal region of the germdisc rather than spreading immediately across the surface (Supporting Figure S2a”'). This static, compacted arrangement prior to expansion resembles the transient serosal configuration described in the honeybee (Fleig and Sander 1986). Later, serosa cells become visible dorsally, appear larger and more morphologically distinct, and begin to move toward the dorsal region (Embedded Video 1 18–25 s). Concurrently, serosa cells begin to undergo epiboly toward the ventral side. As the serosa increasingly stretched, it appeared less distinct on the ventral side; however, epiboly can still be followed by tracking the leading edge (Embedded Video 6). When the trophocytes fully concealed the amnion in the dorsal region, the serosa initiated its expansion posteriorly (Supporting Figure S2b–c”). The serosa advanced as a cap‐like epithelial sheet, enveloping the underlying tissues through a coordinated epiboly from anterior to posterior (Embedded Video 1 18s to 1 min 2 s; Embedded Video 6). Based on live imaging analyses, the serosa often appeared to initiate its expansion earlier in the ventral region, whereas its dorsal progression seemed comparatively slower. However, we cannot exclude the possibility that this observed temporal difference results from embryo‐to‐embryo variation. These movements resulted in a continuous outer layer of serosa with the amnion positioned beneath both the serosa and trophocytes, covering the dorsal yolk. The trophocytes occupied the remaining interstitial space, forming a loosely associated layer between the embryonic and EE domains. Similar to other Hymenoptera, the serosa in *C. floridanus* remained a continuous and intact layer during embryogenesis (Supporting Figure S2d) (Fleig and Sander 1988; Ivanova‐Kasas 1959; Shafiq 1954). Together, our data indicate that the EE tissues act in a coordinated manner during blastoderm stages, with the movements of the serosa and trophocytes occurring in close association with the folding and repositioning of the amnion.

### 2.4 The Argonaute‐Piwi Family Gene *piwi* Marks the Presumptive Amnion in the Early Stages of Its Development

From live imaging, the amnion was observed to slip underneath the germ disc during early embryonic rearrangements (Embedded Video 4). This surprising and unknown morphogenetic movement led us to explore ways to distinguish the amnion tissue boundaries from surrounding tissues better. Although we screened several markers using RNA *in situ* hybridization known to be expressed in the EE tissues in other insects, or expressed in *C. floridanus* embryos generally, we could not identify a reliable amnion marker (Buchta et al. 2013; Rafiqi et al. 2012; Rafiqi et al. 2020). So far, we have tested *Dopa‐decarboxylase* (LOC105254413), *zen* (LOC105251166), *eiger (*LOC105248533), *dpp* (LOC105252168), and *hindsight* (LOC105254215) (data not shown). In an independent screen for germline markers, one of the tested RNA probes for the gene *piwi* stained the presumptive amnion closely in addition to its expected expression in the germline in later stages (Figure 3, Supporting Figure S3). We therefore used *piwi* to follow the amnion tissue.

Along the dorsal side of freshly laid eggs, a pre‐patterned longitudinal, ovate shape was observed, showing *piwi* expression (Supporting Figure S3a,a'). Immediately prior to stage 4, we observed a transversely placed lanceolate shape with *piwi* expression on the dorsal side (Supporting Figure S3b,b'). At stage 4, this transversely positioned lanceolate structure defined by *piwi* expression becomes more distinct, with nuclei now clearly visible at the embryo surface (Figure 3a−d). At this stage, the amnion has not positioned beneath the germ disc yet (Figure 3d'). At early blastoderm stage 5, *piwi* expression begins to extend beneath the germ disc relative to stage 4 (Figure 3e–h), with the leading edge of the amnion clearly visible beneath the germdisc in cross‐sections (Figure 3h'). At late stage 5, the amnion slips further beneath the germ disc and moves posteriorly (Figure 3i–l). In the dorsal view, *piwi* expression extended posteriorly, putatively marking the posterior movement of the amnion (Figure 3i,j). When viewed laterally, *piwi* expression beneath the germ disc becomes more distinct (Figure 3k,l), corresponding to further slipping movement of amnion between the germ disc and yolk (Figure 3l'). When viewed ventrally, *piwi* expression forms a horseshoe‐shaped domain that we interpret as its lateral slipping movement between yolk and germ disc (Figure 3q). From early to late blastoderm stage 5, the amnion adopts a more stretched morphology, consistent with its continued extension and movement. In addition, the *piwi* expression pattern at stage 5 validated the amnion movements seen in live imaging, reflecting expansion along the anterior‐posterior axis and lateral slipping between the germ disc and yolk (Figure 4, Embedded Video 1 2–7 s, and Embedded Video 4). In stage 6, we still detected *piwi* expression beneath the trophocytes in the amnion (Figure 3m–p). However, the leading edge of the amnion is no longer visible beneath the germdisc, suggesting that amnion has completed its inward movement and subsequently relaxed (Figure 3p'). Consistently, the horseshoe‐shaped domain of *piwi* expression is no longer observed in ventral view, supporting this repositioning of amnion (Figure 3r). By stage 7, when the amnion entirely covered the yolk, *piwi* expression was no longer detectable throughout the amnion, although a small amount of expression persisted in the dorsal region (Figure 3s). During late embryogenesis, *piwi* was expressed in the presumptive gonad, consistent with its role in the germline in other insects (Ewen‐Campen et al. 2013; Kawaoka et al. 2008; Megosh et al. 2006; Zhou et al. 2007) (Figure 3t). Together with live imaging, these data confirm that the amnion undergoes dynamic morphogenetic movements during early development.

## 3 Discussion

Our findings in *C. floridanus* ants add to the longstanding framework of synchronous cellularization and direct formation of uniform or differentiated blastoderms (Benton et al. 2013; Donoughe et al. 2022; Handel et al. 2000; Ho et al. 1997; Kelly and Huebner 1989; Nakao 2021; Sarashina et al. 2005; Van Der Zee et al. 2015). We describe a rarely found progressive anterior‐to‐posterior advance of cellularization in *C. floridanus*, which resembles the rare mode of cellularization in *Bombyx mori* (Nakao 2021). However, compared to *B. mori*, *C. floridanus* cellularization from anterior to posterior is coupled with a posterior‐to‐anterior process in the bacteriocyte region. As cellularization progresses, the blastoderm concurrently undergoes regionalization, with distinct domains emerging that correspond to future tissue identities. These features make the *C. floridanus* embryo a variant of differentiating blastoderm organization in which the process of cellularization itself may contribute to early tissue diversification rather than merely partitioning nuclei into cells. Whether and how the presence of endosymbionts in these embryos may have contributed to this mode of cellularization remains to be seen.

In insects, EE tissues exhibit distinct and coordinated morphogenetic movements. In *C. floridanus*, several features of these movements diverge from established insect models. The amnion repositions between the germ disc and the yolk through a slipping movement. While this behavior may superficially resemble folding, our data do not allow us to distinguish between continuous sliding and a transient folded configuration. Notably, at later stages, the amnion is no longer positioned beneath the germ disc, suggesting that this inward configuration is transient. This dynamic repositioning may contribute to the flexibility required for subsequent changes in amnion required for germband movements. The serosa is specified at the anterior region of the germ disc. The boundary between the serosa and the germ disc can be distinguished only when the serosa undergoes a folding process upon itself in the anterior region. Although transient anterior folds have also been described in *Apis mellifera* (Fleig and Sander 1986), the folding behavior observed in *C. floridanus* differs in both its extent and persistence, representing a distinct morphogenetic process. Later, the folds release, and the serosa undergoes epiboly towards the posterior of the egg. This continuous serosa layer stays until the embryo hatches, consistent with other hymenopteran species (Fleig and Sander 1988; Ivanova‐Kasas 1959; Shafiq 1954).

The trophocytes had previously been characterized as serosa in ants (Rafiqi et al. 2020). Cells similar in shape and size can be observed in live imaging snapshots of *M. pharaonis*, although they are not described as such in that species (see Figure 2l–p in (Rajakumar et al. 2024)). However, our data suggest that the serosa and the trophocytes are two distinct cell types, which arise from different locations of the blastoderm, have different morphology, and follow distinct developmental trajectories. This is consistent with earlier descriptions, which describe them as fat cells that are consumed by the embryo in *C. pennsylvanicus* (Tanquary 1913). In many endoparasitoid wasps, a membrane‐like envelope forms around the developing embryo. This structure has been variably termed as serosa, while its cells have been described as teratocytes, trophocytes, or giant cells, depending on their function. These cells have been co‐opted for postembryonic functions wherein they dissociate into autonomously moving ‘teratocytes’ that circulate in the host hemocoel and modulate nutrition, hormones, and immune responses to favor survival of the parasitoid (Dahlman 1990; Danyk and Mackauer 1996; Pedata et al. 2003; Pennacchio et al. 1994; Rouleux‐Bonnin et al. 1999). In *Praon pequodorum*, a parasitoid wasp, in addition to large cells, described as teratocytes or trophocytes, an “extraserosal” envelop has been described, which is positionally analogous to the serosal membrane described for most insects (Danyk and Mackauer 1996). In *C. floridanus*, the serosal envelope covers the trophocytes and persists intact until hatching, whereas the embryo consumes trophocytes during development (Chen et al. 2025). This resemblance between the serosa and extraserosal envelope suggests that trophocytes in *C. floridanus* are similar in position to the teratocytes, whereas what was described as extraserosal in *P. pequodorum* is in fact serosa. If this inference is confirmed, it points to an ancestral developmental potential within hymenoptera for large cuboidal cells underneath the serosal envelope, which either provide a nutritive resource to the embryo or form the teratocyte as in parasitoids, depending on different ecological contexts (Rajakumar et al. 2012). In *C. floridanus*, the trophocytes exhibit strikingly dynamic behaviors and flexibility; as the amnion expands, they are transiently displaced laterally and posteriorly, after which they dorsally enclose the amnion, and the serosa advances and expands over them. These observations suggest that trophocytes cushion the embryonic tissues during morphogenetic processes before serving as nutritive cells.

Our study establishes time‐lapse fluorescent imaging for the ant *C. floridanus*, providing a tool to investigate early development in this system. These results allow us to place the dynamics of cellularization and EE development in *C. floridanus* within a broader comparative framework of insect embryogenesis (Supporting Figure S4). Notably, cellularization proceeds bidirectionally, with opposing fronts moving from anterior to posterior and from the bacteriocyte region toward the anterior, generating an early subdivision of the blastoderm. The early subdivision of the *C. floridanus* blastoderm—wherein anterior regions give rise to both embryonic and EE tissues, while posterior territories accommodate the endosymbiont—may reflect energy prioritization in an endosymbiont‐host context. In this scenario, limited maternal resources could initially be directed towards establishing essential domains, including the germ disc and EE tissues. This may allow the embryo to compartmentalize resource usage and developmental timing across functionally distinct territories for energy‐ and time‐efficient adaptation. This evolved because of endosymbionts, or was an exaptation that allowed the acquisition of the endosymbiont remains to be seen.

## 4 Materials and Methods

### 4.1 Ant Rearing, Embryo Collection, and Fixation

*C. floridanus* colonies were maintained in plastic boxes with glass test tubes filled with water, constrained by cotton wool. They were fed a combination of the Bhatkar–Whitcomb diet (Bhatkar and Whitcomb 1970), cockroaches (*Nauphoeta cinerea*), and 50% honey: water (w/w). All colonies were maintained at 25°C, 50% relative humidity, and a 12‐h day‐night cycle. Queens from different colonies are placed in a separate box with identical colony setups, as mentioned, with 5–10 workers from their colonies. The eggs were collected and developed with workers from the same colony until the desired stage, except for those used for microinjections, which were used immediately after being laid. Fixation was performed according to the previous publication (Khila and Abouheif 2009; Rafiqi et al. 2011; Rothwell and Sullivan 2000). Embryos used for nuclear Hoechst staining and *in situ* hybridization were heat‐fixed using a hot boiling solution of PBS‐Triton (1.86 mM NaH_2_PO_4_, 8.41 mM Na_2_HPO_4_, 1.75 M NaCl, 0.03% Triton‐X‐100, pH 7.4) for 30 s following a 37% formaldehyde‐heptane fixation.

Stages were determined according to Chen et al. (Chen et al. 2025). Briefly, Stage 1 corresponds to freshly laid embryos. Stage 4 was identified by the peripheral positioning of nuclei in the absence of visible cell boundaries. Stage 5 marks the completion of cellularization, resulting in a continuous epithelial layer and the emergence of distinct cell populations. Stage 6 is defined by the onset of ventral mesoderm invagination, followed by stage 7, during which the germdisc elongates to form the germband. Stage 13 corresponds to the end of germband retraction and the onset of dorsal closure.

### 4.2 Phylogenetic Analysis and Gene Cloning

The components of Argonaute/Piwi family genes in the *C. floridanus* genome (Cflo_v7.5 ‐ Genome ‐ Assembly ‐ NCBI (nih. gov)) were identified by TBLASTN (Altschul 1997) using *D. melanogaster* Aubergine, Argonaute, and Piwi protein sequences. Predicted coding sequences of *C. floridanus* were aligned with known Argonaute/Piwi family members from *A. florea*, *A. mellifera*, *D. melanogaster*, *N. vitripennis*, and *T. castaneum* using Translation Align in multiple sequence alignment within Geneious Software (Kearse et al. 2012) (Supporting Table 1). The alignments were refined after exclusion of triplet codon sequences falling within poorly aligned regions. Phylogenetic tree reconstruction was performed with the Neighbor‐Joining method with 1000 bootstrap replicates, without an outgroup. Based on phylogenetic analysis, a single *piwi* ortholog (LOC105251881) was identified in the *C. floridanus* genome (Supporting Figure S5).

For cloning of *Cfl‐piwi*, the total RNA was extracted from mixed‐stage embryos of *C. floridanus* using the TRIzol reagent (Invitrogen) according to the manufacturer's protocol. cDNA was synthesized from total RNA using the Maxima H Minus Reverse Transcriptase (Thermo). A partial fragment of *Cfl‐piwi* coding sequence was amplified from cDNA using gene‐specific primers RL201F (5′‐CGGCCGCGGGAATTCGATTCAC‐3′) and RL201R (5′‐AATCCAACTATCATCAACCCATCC‐3′). The PCR product was purified and cloned into the pGEM‐T Easy vector (Promega) following the manufacturer's instructions. Clones were confirmed by Sanger sequencing (Sanger et al. 1977).

### 4.3 *In Situ* Hybridization

Hybridization Chain Reaction (HCR v3.0) was performed according to the manufacturer's protocol (Choi et al. 2018). Probe sets targeting *Cfl*‐*piwi* were designed using the HCR probe generator tool (Kuehn et al. 2022) and synthesized as probe pools by Integrated DNA Technologies (IDT). A total of 33 probe pairs were used for *Cfl*‐*piwi*. HCR was performed using reagents (hybridization, wash, and amplification buffers, and hairpin amplifiers) obtained from Molecular Instruments. Briefly, embryos were rehydrated, hybridized with probe sets, and subjected to signal amplification using HCR B3 hairpins. Following amplification, samples were passed through a glycerol series (25%, 50%, and 75% glycerol in PBS) before mounting. Hoechst staining was performed at the 25% glycerol step. Samples were then mounted and imaged using a confocal microscope.

Conventional *in situ* hybridization was performed according to previously published methods (Khila and Abouheif 2009; Kosman et al. 2004; Rafiqi et al. 2020). A template was prepared for probe using PCR with M13F and M13R universal primers on the plasmids containing the cloned *Cfl‐piwi* partial fragment. Probe synthesis was performed using SP6 or T7 RNA polymerase (Thermo) according to the supplier's instructions. Alkaline phosphatase secondary antibody anti‐DIG‐AP (Roche) was used to detect DIG‐labeled probes. Probes were purified using the phenol‐chloroform and isopropanol precipitation method, as described in a previous publication (Rafiqi et al. 2020), and were used at a final concentration of 3 ng/μL. Samples were then passed through a glycerol series, mounted as mentioned above, and imaged using a Differential Interference Contrast microscope.

### 4.4 Protein Preparation and Microinjection

The LifeAct–mCherry expression plasmid (a gift from Prof. Steffen Lemke, University of Hohenheim) was transformed into *E. coli* BL21 cells. A single colony was grown overnight in 5 mL Luria−Bertani medium. The next day, the cells were pelleted by centrifugation at 19.5 g for 5 min at 4°C, and washed twice in cold PBS. The pellet containing cells was resuspended in a Tris–EDTA injection buffer (10 mM Tris‐HCl, 1 mM EDTA, pH 7.5) before aliquoting and storage at –80°C. Aliquots were thawed immediately before use, centrifuged at benchtop minispinner, and the supernatant was collected for injection at a constant working dilution of the crude extract, empirically optimized to yield a strong cortical signal while maintaining normal embryo development.

Pre‐cellularization embryos (0–12 h after egg laying), collected from multiple queens, were used for microinjection experiments. Embryos were aligned on a Petri dish lid coated with a thin layer of 1% agar in water supplemented with 10 μl of 10 μg/mL ampicillin to prevent microbial contamination. Embryos were positioned next to a fine glass capillary (Hilgenberg, 0.40 µm). Injection needles were made from borosilicate microcapillaries (WPI_TW100‐4) pulled using a Narishige PC‐100 with the following settings: 81°C heat, 72°C pull, and a two‐step program using a 93 g weight. Injection volumes were adjusted after breaking the tips of the needles, ranging from 0.5 to 1 nL. Embryos were injected using a Narishige microinjection system (IM‐400B suction unit and IM‐400 Electric Microinjector) with 5 psi control pressure, 21 psi injection pressure, and 0.1 s injection duration.

### 4.5 Time‐Lapse Imaging

Live imaging was performed using LifeAct‐mCherry protein injection to label filamentous actin (F‐actin) in the embryos. LifeAct provides a widely used fluorescent marker of F‐actin (Benton et al. 2013; Caroti et al. 2018; Van Der Zee et al. 2015), enabling real‐time observation of furrow ingression and cortical actin organization during cellularization. Only embryos that developed normally after injection were retained for analysis. Different embryos were imaged at the same time from three complementary perspectives—ventral (*n* = 20), dorsal (*n* = 20), and lateral (*n* = 20)—each of which revealed distinct features of cellularization and EE tissue organization. Injected embryos were incubated for approximately 2 h at room temperature for recovery, then mounted in halocarbon oil 700 (H8898) and imaged on a Leica SP8 confocal microscope with a 20X objective. Time‐lapse recordings were acquired every 10 min at 20°C ± 1°C and 50% humidity, covering 30–40 z‐sections at 2 µm step size. Cell morphology and cortical actin distribution were analyzed from the LifeAct‐mCherry signal. The injections were only successful when performed in the syncytial stage, and fluorescent protein was detectable for a maximum of 72 h post‐injection. Due to these limitations, morphogenetic movements could be reliably followed until the serosa fully envelops the embryo.

### 4.6 Microscopy and Image Analysis

We used a Leica Differential Interference Contrast microscope, along with Leica software, to image embryos for conventional in situ hybridization. For high‐resolution imaging, a Leica SP8 confocal microscope was employed for live imaging, as well as for imaging Hoechst‐stained embryos and embryos processed by HCR. For quantitative and image analyses, ImageJ2 software was utilized (Rueden et al. 2017). To pseudocolor different tissues, RGB images were split into individual R, G, and B channels using the ‘Split Channels’ function. Regions of interest corresponding to specific cell popuflations were manually selected using the selection tools and removed. After the editing steps, the channels were recombined using ‘Merge Channels’ to generate the final pseudocolored composite image.

## Author Contributions

Nihan Sultan Milat and Ab. Matteen Rafiqi conceived the idea, Nihan Sultan Milat conducted the experiments and analysis. Murat Pekmez assisted in data analysis. Nihan Sultan Milat and Ab. Matteen Rafiqi wrote the paper.

## Conflicts of Interest

The authors declare no conflicts of interest.

## Supporting information

Supporting File 1

Supporting File 2

Supporting File 3

Supporting File 4

Supporting File 5

Supporting File 6

Supporting File 7

Supporting File 8

## Data Availability

The data that supports the findings of this study are available in the supporting material of this article.
