# Supplementary material for: Progressive Cellularization of Blastoderm and Extraembryonic Tissue Formation in the Ant Camponotus floridanus
Source: J Exp Zool B Mol Dev Evol. 2026 Jun 14;346(5):411–21. doi: 10.1002/jez.b.70028 (PMC13313101; doi:10.1002/jez.b.70028)
Supplement: Supplementary file 1 — Supporting File 1 [file JEZ-346-411-s006.docx]

**Supporting Figure 1**

**
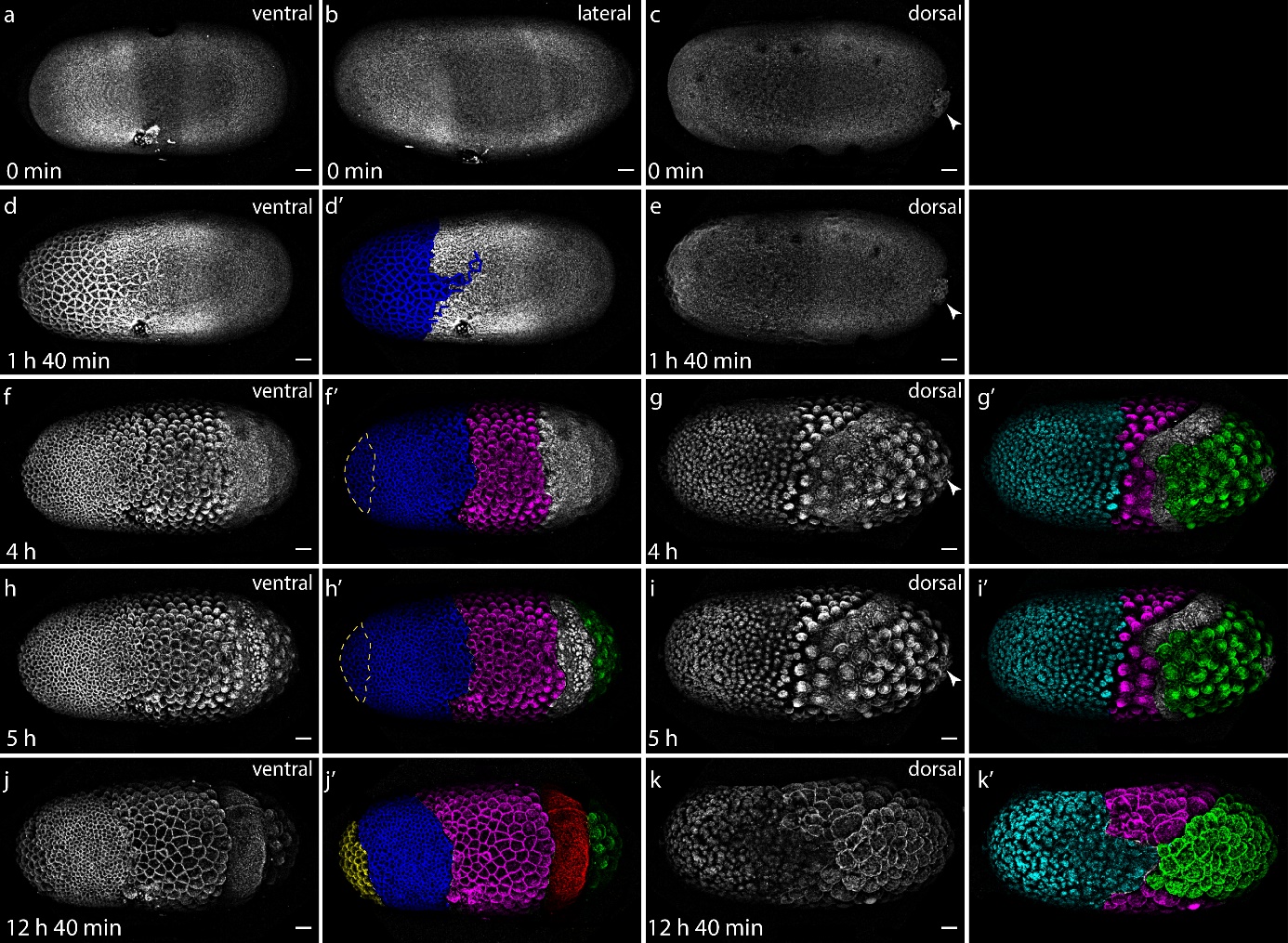
**

**Supporting Figure 1: Representative images of *C. floridanus* embryos at the blastoderm stage.**

**(a-k’)** Additional representative images corresponding to Figure 2, showing the blastoderm stage. (a-c) Embryos showing pre-cellularization. **(d-k’)** Embryos showing cellularization and formation of tissues over time during the blastoderm. Corresponding views are color-coded for clarity. The serosa (yellow), germdisc (blue), amnion (cyan), trophocytes (magenta), germline capsule (red), and bacteriocytes (green) are indicated. Each row represents the same embryo imaged from the ventral and dorsal orientations, except **(b)** is lateral. Each panel represents a different embryo imaged from a distinct orientation. White arrowheads indicate pole cells. The yellow dashed lines indicate the approximate region where presumptive serosa forms. Anterior is to the left, and dorsal is up. Scale bar = 50 µm.

**Supporting Figure 2**

**
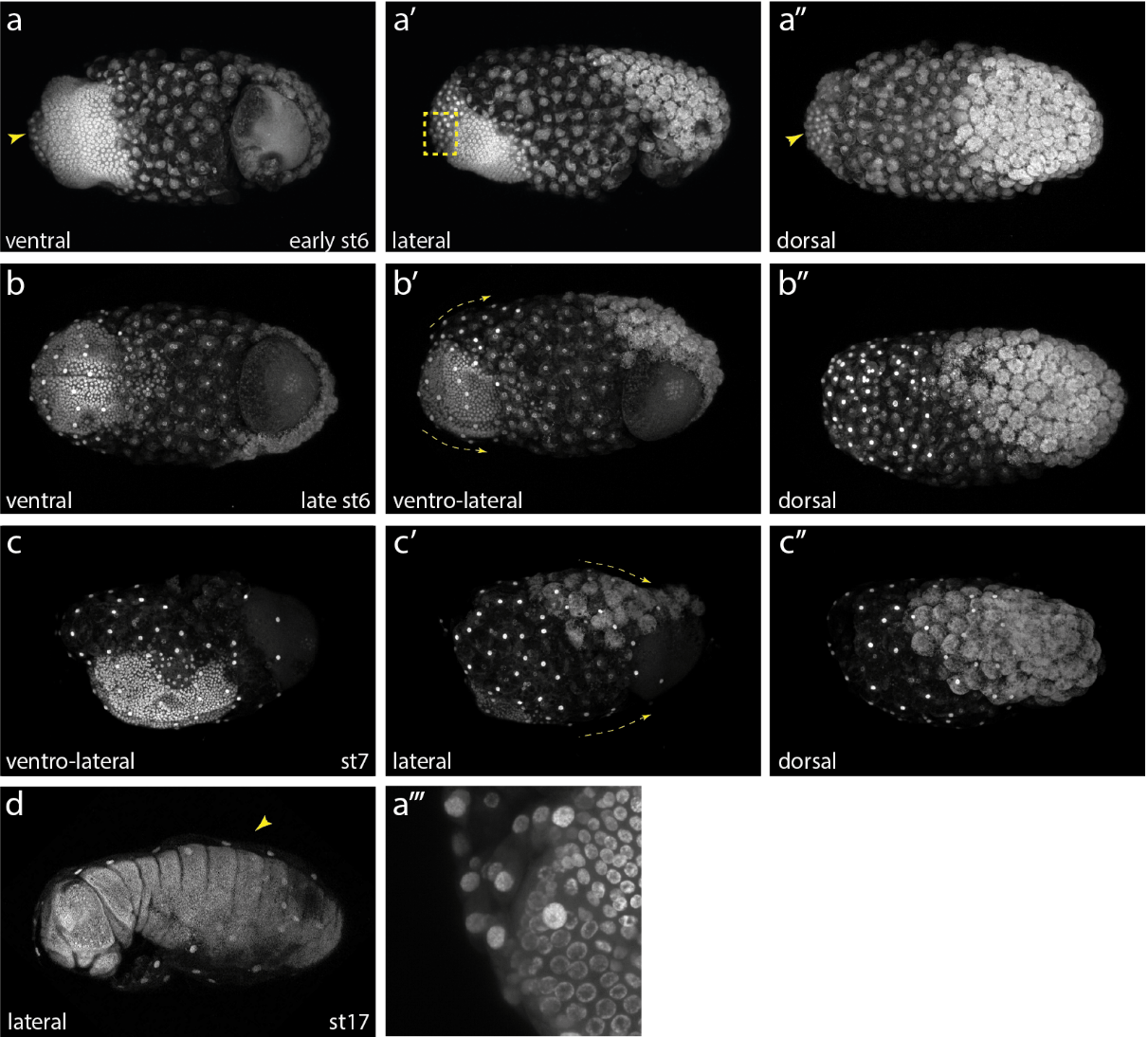
**

**Supporting Figure 2: Serosa formation and epiboly in different embryonic stages in *C.
floridanus*.**
**(a-d)** Confocal scanned images of Hoechst-stained embryos. **(a-a”)** early stage 6, **(b-b”)** late stage 6,and **(c-c”)** stage 7, and **(i)** stage 17. Yellow arrowheads indicate serosa and dashed lines indicate epiboly of serosa. Note that the embryonic nuclei and the serosa nuclei
differ in morphology, noticeable in confocal scan (a’’’). Anterior is to the left, dorsal is to the top.

**Supporting Figure 3**

**
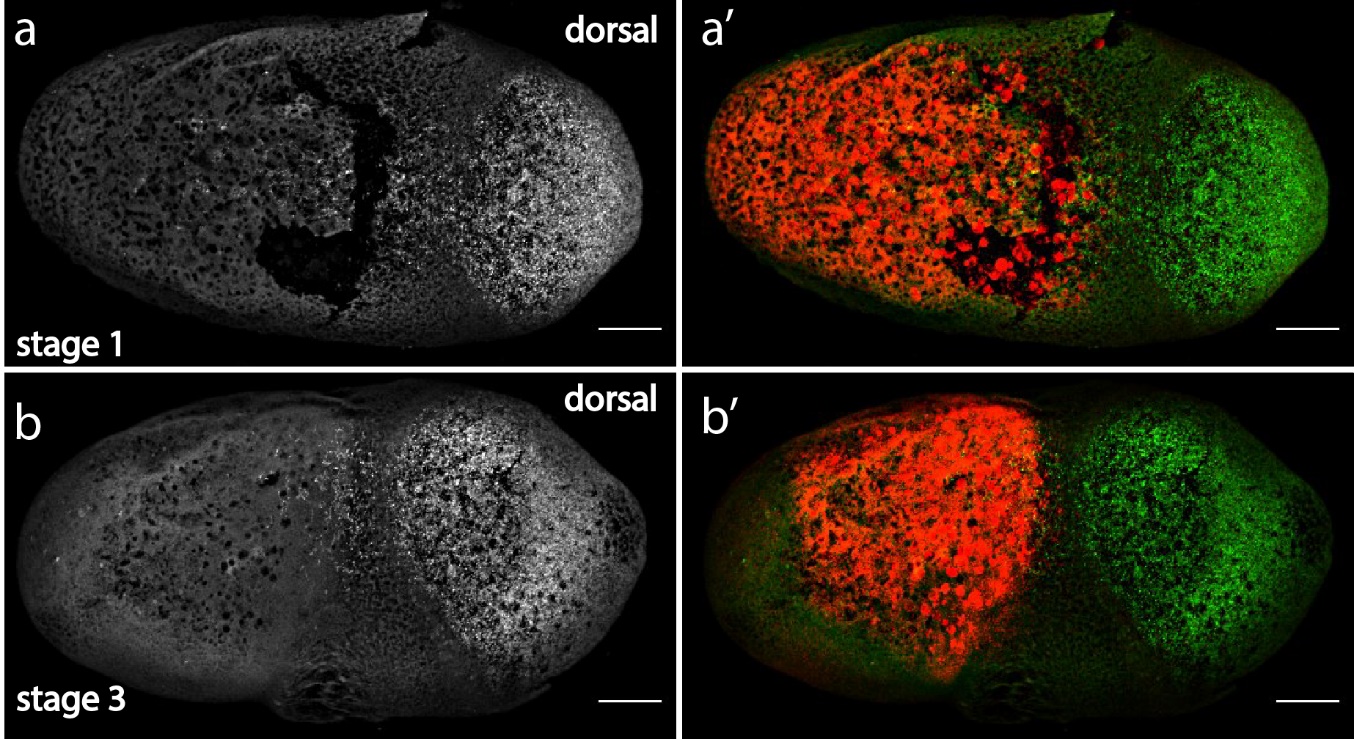
**

**Supporting Figure 3: Cfl-piwi expression marks the region of the prospective amnion in early embryos**

**(a-b)** Confocal-imaged *C. floridanus* embryos stained with a probe against *Cfl-piwi*. **(a)** freshly laid egg, Stage 1 and **(b)** Stage 3**.** Note that confocal-imaged embryos are stained with Hoechst (a-b) and shown in grayscale, except in merged panels where embryo is shown in green and *piwi* expression in red (a’ and b’). Fixed embryos were stage-matched based on morphology according to (*Chen et al., 2025*). Anterior is to the left.

Chen, T., Rafiqi, A. M., Rajakumar, A., Milat, N. S., Lamouret, T., Plante, É., Fung, B. A., Rajakumar, R., & Abouheif, E. (2025). A Developmental Table for the Florida Carpenter Ant *Camponotus floridanus*: Establishing Foundations for Mechanistic Studies of Development and Evolution in Ants. *Qeios*. <https://doi.org/10.32388/OJQC0N>

**Supporting Figure 4**

**
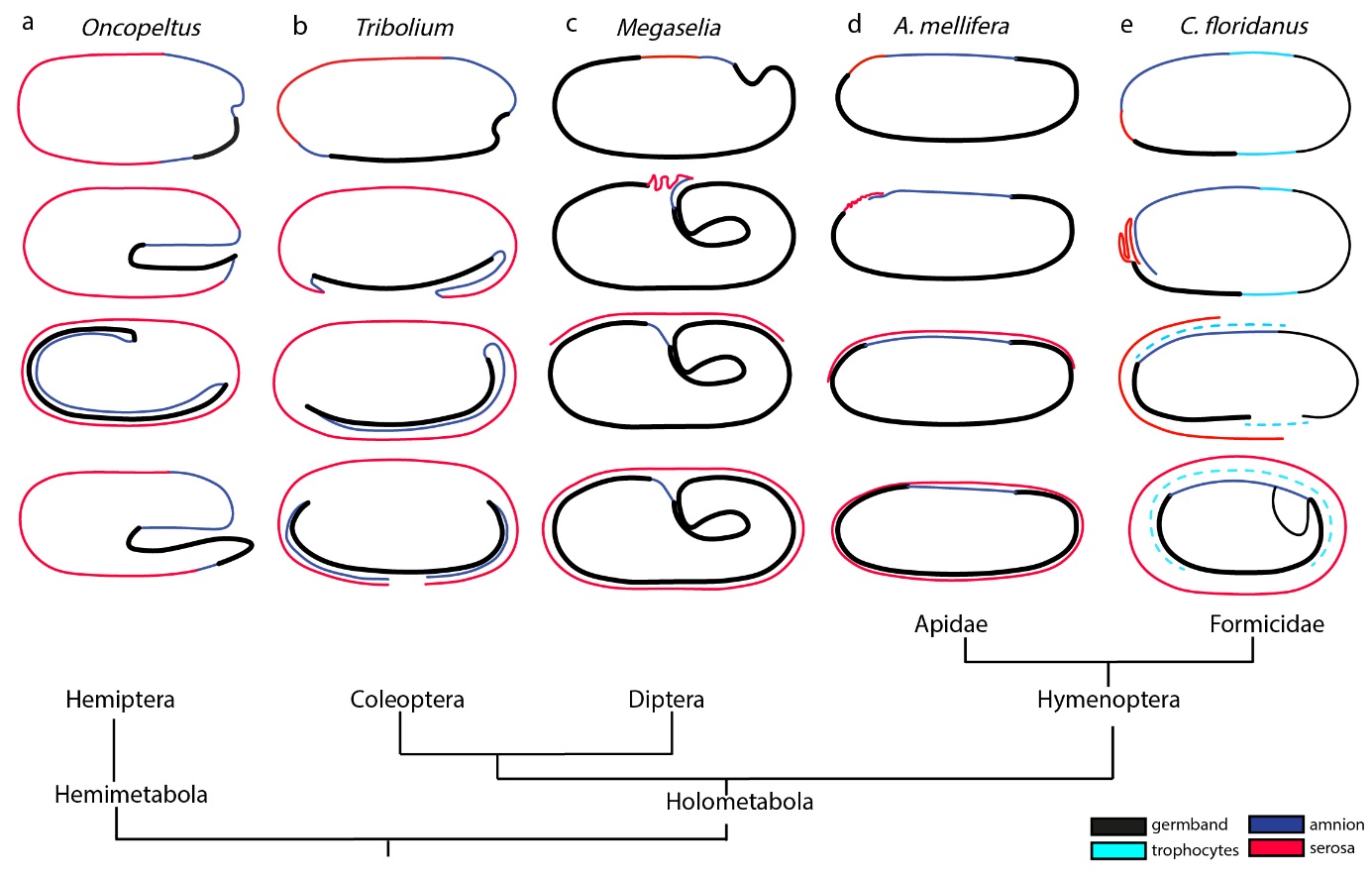
**

**Supporting Figure 4: Comparison of extraembryonic development in insects.**

Schematics of embryos highlighting the formation and positioning of extraembryonic tissues in representative insects (a) *Oncopeltus fasciatus*, (b) *Tribolium castaneum*, (c) *Megaselia abdita*, (d) *Apis mellifera*, (e) *Camponotus floridanus*. Anterior is to the left, and dorsal is up. Different colors indicate different tissues. Dashed lines indicate discontinuous tissues, while solid lines indicate continuous tissues. Modified from (*Rafiqi et al., 2008; Schmidt-Ott & Kwan, 2016*)

Rafiqi, A. M., Lemke, S., Ferguson, S., Stauber, M., & Schmidt-Ott, U. (2008). Evolutionary origin of the amnioserosa in cyclorrhaphan flies correlates with spatial and temporal expression changes of zen. *Proceedings of the National Academy of Sciences*, *105*(1), 234-239.

Schmidt-Ott, U., & Kwan, C. W. (2016). Morphogenetic functions of extraembryonic membranes in insects. *Current opinion in insect science*, *13*, 86-92.

**Supporting Figure 5**

**
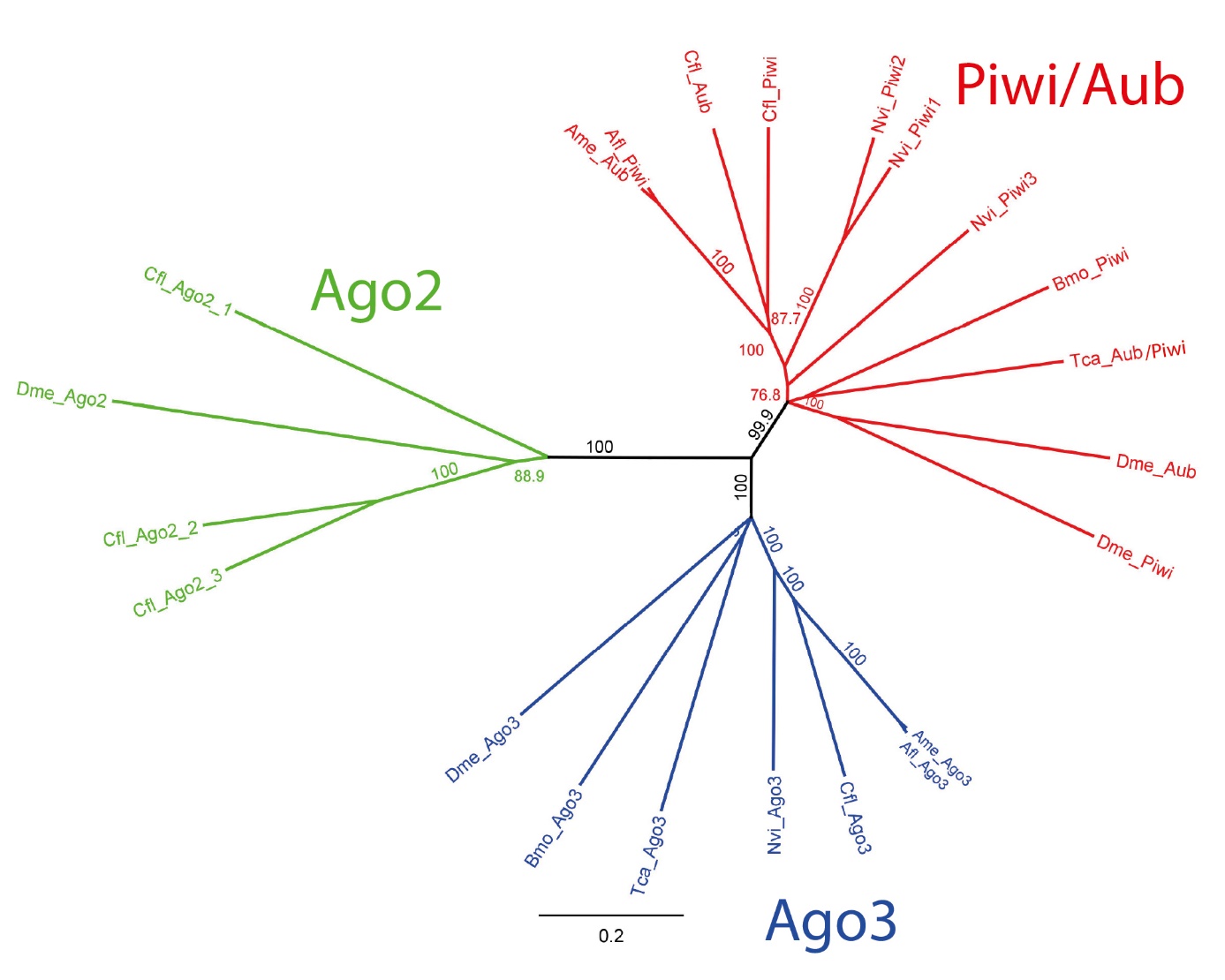
**

**Supporting Figure 5: Orthologies of Aubergine, Argonaute*,* and Piwi inferred by the Neighbor-Joining method.**

The phylogenetic tree was constructed using the Neighbor-Joining method with 1,000 bootstrap replicates; bootstrap support values are shown as percentages at the nodes. Orthologs of Aubergine, Argonaute, and Piwi were identified through BLAST-based searches and aligned using Translation Alignment in Geneious from *C. floridanus, Apis florea, Apis mellifera, Nasonia vitripennis, Drosophila melanogaster, Tribolium castaneum, and Bombyx mori.* Phylogenetic analysis follows previous studies (*Liao et al., 2010; Lu et al., 2011; Rubio et al., 2018; Tomoyasu et al., 2008*) .

Liao, Z., Jia, Q., Li, F., & Han, Z. (2010). Identification of two piwi genes and their expression profile in honeybee, Apis mellifera. *Archives of Insect Biochemistry and Physiology: Published in Collaboration with the Entomological Society of America*, *74*(2), 91-102.

Lu, H.-l., Tanguy, S., Rispe, C., Gauthier, J.-P., Walsh, T., Gordon, K., Edwards, O., Tagu, D., Chang, C.-c., & Jaubert-Possamai, S. (2011). Expansion of genes encoding piRNA-associated argonaute proteins in the pea aphid: diversification of expression profiles in different plastic morphs. *PloS one*, *6*(12), e28051.

Rubio, M., Maestro, J. L., Piulachs, M.-D., & Belles, X. (2018). Conserved association of Argonaute 1 and 2 proteins with miRNA and siRNA pathways throughout insect evolution, from cockroaches to flies. *Biochimica et Biophysica Acta (BBA)-Gene Regulatory Mechanisms*, *1861*(6), 554-560.

Tomoyasu, Y., Miller, S. C., Tomita, S., Schoppmeier, M., Grossmann, D., & Bucher, G. (2008). Exploring systemic RNA interference in insects: a genome-wide survey for RNAi genes in Tribolium. *Genome biology*, *9*(1), R10.
